# Dehydrocostus lactone inhibits cell proliferation and induces apoptosis by PI3K/Akt/Bad and ERS signalling pathway in human laryngeal carcinoma

**DOI:** 10.1111/jcmm.15131

**Published:** 2020-04-21

**Authors:** Ren Zhang, Ji Hao, Qingming Wu, Kaiwen Guo, Chao Wang, Wei Kevin Zhang, Wanxin Liu, Qiang Wang, Xinzhou Yang

**Affiliations:** ^1^ Institute of Infection, Immunology and Tumor Microenviroment, Hubei Province Key Laboratory of Occupational Hazard Identification and Control Medical School Wuhan University of Science and Technology Wuhan China; ^2^ School of Pharmaceutical Sciences South‐Central University for Nationalities Wuhan China

**Keywords:** apoptosis, cytotoxicity, dehydrocostus lactone, endoplasmic reticulum stress, laryngeal carcinoma, PI3K/Akt/Bad, *Saussurea costus*

## Abstract

The anti‐cancer effect of dehydrocostus lactone (DHL) derived from *Saussurea costus* (Falc.) Lipech against laryngeal carcinoma was assessed. The cytotoxic activity of DHL against laryngeal carcinoma is still obscure. Therefore, our study investigated the role of DHL in the growth inhibition of laryngeal carcinoma in vitro and in vivo, and the molecular mechanism of DHL‐induced apoptosis in cancer cells of the larynx. The results showed that DHL inhibits the viability, migration and proliferation of Hep‐2 and TU212 cells with little toxic effects on human normal larynx epithelial HBE cell line. Flow cytometry analysis (FAC) analysis and staining assay (Hoechst 33258) indicated that DHL stimulated Hep‐2 and TU212 cell apoptosis in a dose‐dependent manner. Mechanistically, DHL is capable of inhibiting Hep‐2 and TU212 cell viability via promoting p53 and P21 function, meanwhile DHL dose‐dependently induces Hep‐2 and TU212 cells apoptosis via activating mitochondrial apoptosis by inhibiting PI3K/Akt/Bad pathway and stimulating endoplasmic reticulum stress‐mediated apoptosis pathway. In vivo, DHL inhibited the growth of the Hep‐2 nude mouse xenograft model and observed no significant signs of toxicity in the organs of nude mice. In vivo experiments further confirmed the anti‐cancer effect of DHL on laryngeal carcinoma cells in vitro, and DHL‐treated nude mice can reduce the volume of tumours. Together, our study indicated that DHL has the potential to inhibit human laryngeal carcinoma via activating mitochondrial apoptosis pathway by inhibiting PI3K/Akt/Bad signalling pathway and stimulating endoplasmic reticulum stress‐mediated apoptosis pathway, providing a strategy for the treatment of human laryngeal carcinoma.

## 1 INTRODUCTION

With the development of industry and the aggravation of air pollution, the risk of cancer is increasing.^1^ Therefore, effective methods are urgently needed to fight against cancer. Laryngeal carcinoma is one of the most common tumours of head and neck, with the morbidity ranking second among all respiratory tract tumours.^2^, ^3^ Chemotherapy and radiation are not effective treatments for laryngeal cancer, and overall survival rates have not been improved, according to new research.^4^, ^5^ The development of new anti‐cancer agents or new therapeutic strategies to enhance the sensitivity of laryngeal cancer cells to radiotherapy and chemotherapy is one of the most effective ways to treat patients with laryngeal cancer.

Traditional Chinese medicine has a wide range of pharmaceutical potential, as well as over thousands of anti‐cancer plants with low toxicity and high efficiency.^6^ Therefore, the discovery of natural drugs from traditional Chinese medicine against laryngeal cancer has become our primary research goal. Our investigation found that dehydrocostus lactone (DHL) derived from *Saussurea costus* (Falc.) Lipech has potential anti‐cancer activity on various types of cancers, which has attracted our attention and interest in this compound. DHL achieves an anti‐ovarian cancer effect by inhibiting the cell cycle distribution of ovarian cancer cells and inducing apoptosis.^7^ It inhibits the proliferation of liver cancer cells through intrinsic apoptotic pathway and exerts anti‐cancer effects.^8^ The compounds induce apoptosis of non‐small‐cell lung cancer cells through oxidative and endoplasmic reticulum stress signalling pathways,^9^ and DHL induces prostate cancer cell apoptosis through the mitochondrial pathway to inhibit prostate cancer cell proliferation.^10^ The above‐mentioned experimental studies on the anti‐cancer effect of DHL have fully proved that the compound is a potential anti‐cancer agent. In addition, DHL also has antifungal,^11^ anti‐inflammatory,^12^ antiviral,^13^ antiulcer,^14^ antioxidant^15^ and antidiabetic effects.^16^ However, there are few reports on the cytotoxicity of DHL for laryngeal carcinoma cells, and the molecular mechanism by which DHL induces apoptosis in laryngeal carcinoma is unclear. In our study, we aim to explore the anti‐cancer effects of DHL on human laryngeal carcinoma, and study the undiscovered mechanism of action of DHL on human laryngeal carcinoma.

In this study, dehydrocostus lactone (DHL), a natural sesquiterpene lactone, was purified from the plant species *Saussurea costus* (Falc.) Lipech. Further anti‐proliferative assay showed that DHL inhibited proliferation of laryngeal carcinoma cells in a time‐ and dose‐dependent manner, but showed little cytotoxicity in the epithelial cells of human larynx. Further, we also revealed that DHL had the capacity to inhibit migration of TU212 and Hep‐2 cells, as well as to provoke laryngeal carcinoma cells apoptosis. Mechanistically, DHL inhibits the proliferation of laryngeal carcinoma cells by controlling the process of cell cycle, meanwhile DHL dose‐dependently induced apoptosis of laryngeal carcinoma cells via activating mitochondrial apoptosis pathway by inhibiting PI3K/Akt/Bad signalling pathway and stimulates endoplasmic reticulum stress‐mediated apoptosis.

## 2 MATERIALS AND METHODS

### 2.1 Plant material

The roots of *Saussurea costus* (Falc.) Lipech (family Compositae) were collected from Wufeng County, Hubei province, China in July, 2015, and identified by Professor Dingrong Wan of School of Pharmaceutical Sciences, South‐Central University for Nationalities (SCUN), Wuhan, China. A voucher specimen (No. SC0691) was deposited in School of Pharmaceutical Sciences, SCUN, Wuhan, China.

### 2.2 Chemicals and reagents

High‐performance liquid chromatography (HPLC)‐grade solvents were used for chromatography, and all other chemicals were of analytical reagent grade. HPLC‐grade acetonitrile (MeCN) and methanol were purchased from Tedia Company. Sephadex LH‐20 gel was obtained from GE Health Care. Dulbecco's modified Eagle's medium (DMEM), foetal bovine serum (FBS) and antibiotics (100 U/mL penicillin and 100 µg/mL streptomycin) were obtained from Hyclone. Annexin V‐FITC kit and PI kit were purchased from BD Pharmingen. CCK‐8 was obtained from Sigma. caspase‐3(9962), caspase‐9(9508), Bax (5023), Bad (9268), Bcl‐2 (2870), cyclin D1 (2978), CHOP (2895), PARP (9542), PTEN (9559), Akt (4691), Phospho‐Akt (Ser473) (4060),Phospho‐Bad (Ser136) (4366), p53 (2524), p21 Waf1/Cip1 (2947), MMP‐2 (40994), MMP‐9 (13667) and β‐actin (3700) were purchased from Cell Signaling Technology. Caspase‐12 (GTX132298) and Grp‐78/Bip (GTX113340) were purchased from GeneTex.

### 2.3 General experimental procedures

Semi‐preparative HPLC was carried out on a Waters 2535 HPLC fitted with a 2998 Photodiode Array Detector and a 2707 Autosampler (Waters). Separations were performed on Thermo C18 columns (5 µm, 10 × 250 mm; 5 µm, 20 × 150 mm). EIMS data were obtained with MAT‐95 mass spectrometer. NMR spectra were recorded on an AVANCE III 600 MHz spectrometer (Bruker BioSpin).

### 2.4 Extraction and isolation

Air‐dried roots of *Saussurea costus* (2.0 kg) were smashed and extracted by maceration at room temperature with 80% ethanol (4 × 5 L, 3 days each). The solvents were evaporated at reduced pressure to yield 350 g of residue. The residue was dismissed to slurry by water (1:10), and the slurry was then extracted with petroleum ether (4 × 2.5 L). The solvents were evaporated at reduced pressure to yield petroleum ether extract (60 g). The petroleum ether extract (57 g) was subjected to D101 macroporous resin column chromatography (CC) (600 g, Sinopharm Chemical Reagent Co., Ltd.) eluted with 20%, 40%, 60%, 80% and 95% aq. ethanol in a gradient manner. The crystal (10 g) was filtered from 80% aq. ethanol eluted fraction, and was determined as DHL by comparing its ^1^H‐NMR, ^13^C‐NMR, mass spectroscopical data, Optical Rotation and ROESY (Figures S1‐S5) with those reported in the literatures.^17^, ^18^, ^19^

### 2.5 Chemical elucidation of DHL

Dehydrocostus lactone (DHL): C_15_H_18_O_2_, yellow powder; [α]‐16.4° (*c* 1.00, CHCl_3_); EI‐MS: *m/z* 230 [M]^+^. ^1^H‐NMR (600 MHz, CDCl_3_): *δ*_H_ 6.22 (1H, d, *J* = 3.5 Hz, H‐13α), 5.49 (1H, d, *J* = 3.5 Hz, H‐13*β*), 5.27 (1H, d, *J* = 1.8 Hz, H‐15*α*), 5.07 (1H, d, *J* = 1.8 Hz, H‐15*β*), 4.90 (1H, br s, H‐14*α*), 4.81 (1H, br s, H‐14*β*), 3.96 (1H, t, *J* = 9.2 Hz, H‐6), 2.83‐2.95 (3H, m, H‐1, 5, 7), 2.46‐2.60 (3H, m, H‐3*α*, 3*β*, 9*α*), 2.24 (1H, m, H‐8*α*), 2.16 (1H, m, H‐9*β*), 1.95 (1H, m, H‐2*α*), 1.87 (1H, m, H‐2*β*), 1.42 (1H, m, H‐8*β*). ^13^C‐NMR (150 MHz, CDCl_3_): 170.4 (C‐12), 151.4 (C‐4), 149.4 (C‐10), 139.9 (C‐11), 120.3 (C‐13), 112.8 (C‐14), 109.8 (C‐15), 85.4 (C‐6), 52.2 (C‐5), 47.8 (C‐1), 45.3 (C‐7), 36.4 (C‐9), 32.7 (C‐3), 31.1 (C‐8), 30.5 (C‐2).^1^H‐NMR, ^13^C‐NMR, mass spectroscopical data, Optical Rotation and ROESY of DHL could be found in Supporting Information.

### 2.6 Cell culture

Hep‐2 cell line was purchased from the American Type Culture Collection (ATCC). TU212 and HBE cell line were obtained from the Shanghai Institute of Biochemistry and Cell Biology, Chinese Academy of Sciences (Shanghai, China). The cells were cultured in Dulbecco's modified Eagle's medium (DMEM; Sigma‐Aldrich) supplemented with 10% (v/v) heat‐inactivated foetal bovine serum (Sigma‐Aldrich) and 1% penicillin/streptomycin (Hyclone) at 37°C in a humidified atmosphere of 5% CO_2_. DHL was dissolved in dimethyl sulphoxide (DMSO, MP bio) and stored at −20°C for until use. The final in‐well DMSO concentration in cells is <0.1% (v/v).

### 2.7 Cell viability analysis

Cell Counting Kit‐8 (Beyotime) assays were used to evaluate the cell growth inhibitory effect of DHL. Hep‐2, TU212 and HBE cells were seeded at 1 × 10^5^ cells/well into 96‐well plates and allowed to grow for culture to 80% per well. Before treatment, DHL was dissolved in DMSO as stock solution and diluted by serum‐free DMEM to various concentrations (0, 2, 4, 6, 8 and 10 μg/mL). Hep‐2 and TU212 cells were respectively incubated with different concentrations of DHL for 6, 12, 24 hours. Then, absorbance in each well was measured at 450 nm by a spectrophotometer (Thermo Scientific). Calculating the inhibition rate uses the following formula: inhibition rate (%) = [1 − (ODsample − ODblank)/(ODcontrol − ODblank)] × 100. The IC_50_ value of DHL is defined as the concentration at which cell inhibition rate was 50% as determined by CCK‐8 assays and calculated by the GraphPad Prism 6.0 Software.

### 2.8 Wound healing assay

Hep‐2 and TU212 cells were seeded at 4 × 10^5^ cells/well into 12‐well plates and allowed to grow for about 24 hours until confluence. After respectively incubated with 1.0 mL of DHL (0, 3 μg/mL), scratch wounds were photographed immediately as 0 hour. Then scratch wound images of the same field were photographed at 12, 24 hours after scratch under a phase contrast microscope (Leica). The measurement of wound surface area was calculated with ImageJ software.

### 2.9 Observation of morphological changes and Flow Cytometry Analysis (FACs)

Hep‐2 and TU212 cells were seeded at 1 × 10^5^ cells/well in 6‐well plates and allowed to grow for 12 hours. Cells were respectively incubated with 2.0 mL of DHL (2, 4 and 6 μg/mL) and CDDP (10 µg/mL) for 12 hours; DMSO‐treated group served as control, and CDDP‐treated group served as positive group. After cultivation, the cellular morphological changes were observed and photographed at a magnification of 80× under a phase contrast microscope (Leica). Then, the cells were immobilized and stained by Hoechst 33258 (10 μg/mL). Subsequently, the cellular nuclear morphological changes were observed and photographed at a magnification of 80× under a fluorescence microscope (Leica). Hep‐2 and TU212 cells were preprocessed, and FACS was performed as the protocol described. Cells fluorescence was then assayed by flow cytometry (Guava easyCyte) and analysed by FlowJo software. Qualitative observation was performed by fluorescence microscopy.

### 2.10 Western blot analysis

Hep‐2 and TU212 cells were seeded at 2 × 10^5^ cells/well into 100 × 20 mm cell culture dish and allowed to grow for 12 hours. Cells were respectively incubated with 9.0 mL of DHL (0, 2, 4 and 6 μg/mL). After cultivation, cells were collected and lysed in RIPA containing phenylmethanesulphonyl fluoride and PhosSTOP (Nantong, Jiangsu Province, China). Insoluble cell debris was discarded following centrifugation (12 000*g*) at 4°C for 30 minutes. The supernatant containing protein was collected and stored at −80°C until use. The measurement of protein content was performed with a BCA kit (Nantong, Jiangsu Province, China). Cell lysates were separated by SDS‐polyacrylamide gel electrophoresis (SDS–PAGE) on 7.5%‐12.5% gels and then transferred onto polyvinylidene membranes (Bio‐Rad). And then the membranes were blocked by 5% skim milk in tris‐buffered saline with tween 20 (0.5%) for 2 hours. Subsequently, the membranes were incubated with the primary antibody at 4°C overnight and incubated with the secondary antibody at 37°C for 2 hours. The HRP ECL system (Nantong, Jiangsu Province, China) was used to detect the protein band, and the film was scanned and saved.

### 2.11 Immunohistochemical examinations of transplanted tumour tissues (IHC)

Immunohistochemical examinations were conducted as described previously.^20^ Briefly, after embedding in paraffin, xenografts were cut into sections and incubated with the indicated primary antibodies (caspase‐3, Grp78, PTEN and p‐Akt) and visualized by corresponding secondary antibodies conjugated with horseradish peroxidase. After staining and sealing, photographs were taken at the magnification of 200×. Positive staining was quantified by Image‐Pro Plus 6.0 software.

### 2.12 Immunofluorescence

Immunofluorescence staining was performed on tumour sections, sections were rinsed with PBS, followed by blocked with 5% nonspecific antigen goat serum for 1 hour at 37°C. Then, the sections were incubated overnight at 4°C with specific primary antibodies (CHOP). In the next day, after rinsed with PBS containing 0.5% Triton X‐100 for three times, sections were incubated for 1 hour at 37°C with corresponding secondary antibodies. The primary and secondary antibodies were diluted in PBS containing 5% normal goat serum and 0.2% Triton X‐100. After incubation with secondary antibodies, the sections were mounted on glass slides and cover glasses were slipped in mounting medium. Fluorescent images were captured with a confocal microscope (LSM880, Zeiss).

### 2.13 Terminal Deoxynucleotidyl Transferase dUTP Nick End Labelling (TUNEL)

Xenografts were embedded in paraffin and cut into sections (5 µm) after pretreatment with ice‐cold saline and fixed in buffered neutral 10% formalin. The sections were subjected to TUNEL assay by TUNEL assay kit (Roche Biotechnology) according to the manufacturer's instructions. After staining and sealing, photographs were taken at the magnification of 200×. Apoptosis index was quantified by the yellow‐stained apoptotic nucleus. Ten regions were chosen from the photographs of tumour sections randomly, then blinded and counted by two people. Their mean values were used in statistical analysis. To avoid the discrepancy between two observers, a datum was valid only if the discrepancy between the two observers was <10%.

### 2.14 Animal studies

Female nude mice (BALB/c nu/nu, 4‐5 weeks old, 18‐19 g) were purchased from the Beijing HFK Bioscience Co, Ltd (SCXK 2014‐0004). Briefly, 150 µL of Hep‐2 cells (1 × 10^7^ per mouse) was subcutaneously transplanted into the right flank of the nude mice. When tumours reached 100 mm^3^, the tumour cell‐inoculated mice were randomly divided into the following three treatment groups (A‐C groups) with five mice in each group. Group A was treated with propylene glycol (PG), group B was treated with 10 mg/kg DHL, and group C was treated with 15 mg/kg DHL. All treatments were given intraperitoneal injection for 2 days. The tumours were measured using a caliper every 4 days, and the tumour volume was calculated according to the formula V = 1/2 (width^2^ × length). Three weeks later, mice were killed and transplanted tumours were removed for assessments. All procedures were in accordance with the National Institutes of Health Guide for the Care and Use of Laboratory Animals (National Institutes of Health, Bethesda, MD, USA). The protocol was approved by the Animal Care and Use Committee of South‐Central University for Nationalities (Wuhan, China).

### 2.15 Statistical analysis

All data are shown as mean ± SD from three independent experiments. GraphPad Prism 6.0 software was used for analysis. Statistically, differences were analysed by one‐way analysis of variance (ANOVA), and *P*‐values < .05 were considered significant.

## 3 RESULTS

### 3.1 DHL inhibits proliferation of laryngeal carcinoma cells

**Figure 1:**
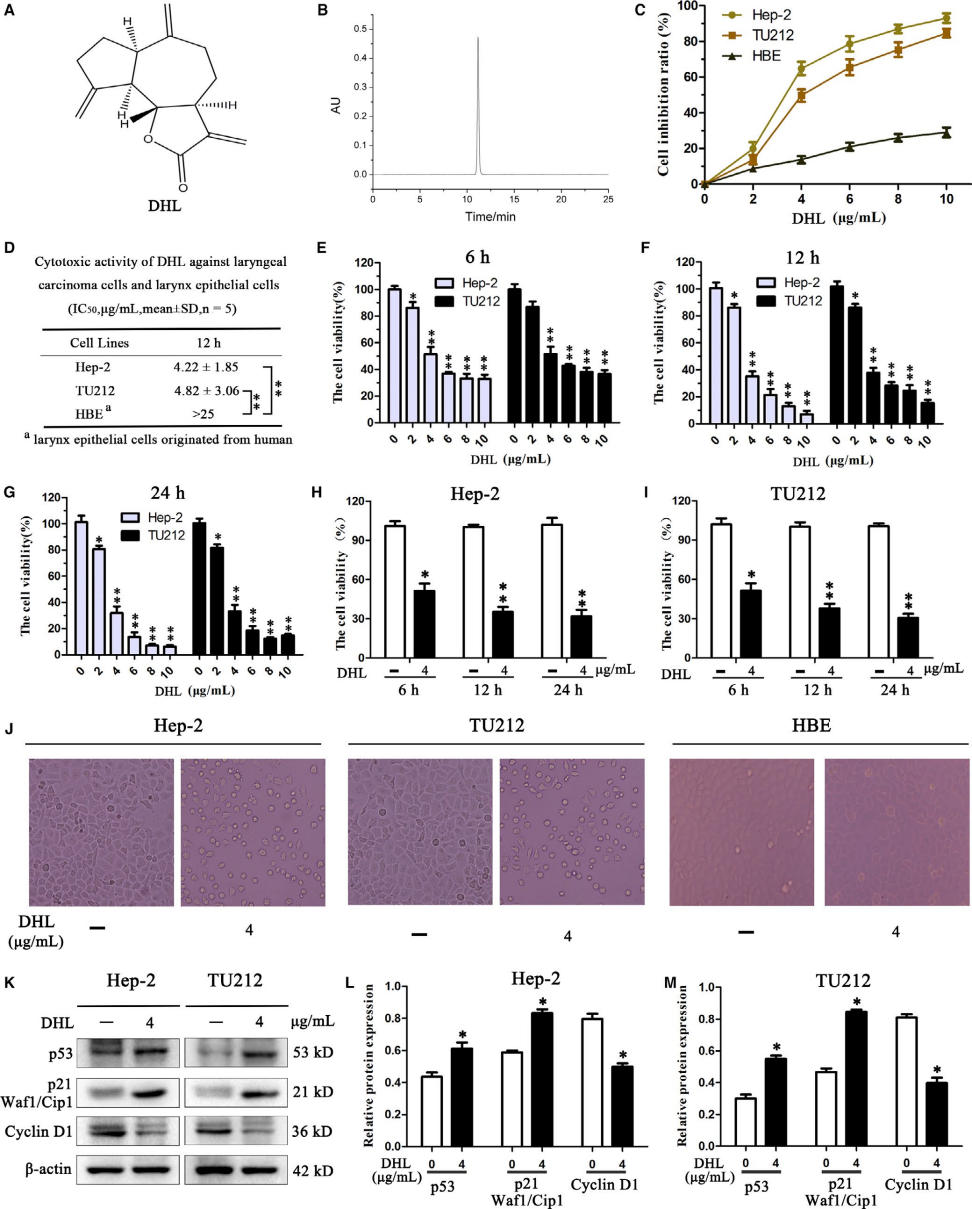
Dehydrocostus lactone (DHL) inhibited proliferation of laryngeal carcinoma cells. A, Chemical structure of DHL. B, HPLC chromatogram of DHL. C, Hep‐2, TU212 and HBE cells were incubated with 0, 2, 4, 6, 8 and 10 μg/mL of DHL for 12 h. D, For each cell line, IC_50_ values were determined. E‐G, Treatment of Hep‐2 and TU212 cells was done with specified concentrations of DHL. Cell viability was examined after 6, 12, 24 h by CCK‐8. H and I, Hep‐2, TU212 cells were incubated with 4 μg/mL of DHL for 6, 12, 24 h. J, Morphological observation of Hep‐2, TU212 and HBE cells after DHL treatment. DHL at 4 μg/mL induced laryngeal carcinoma cell shrinkage, distortion and disengage of cellular matrix without obvious cytotoxicity on HBE cells. K‐M, The effects of DHL on p53, p21 and cyclin D1 protein. These proliferation‐associated proteins were measured by Western blot in TU212 and Hep‐2 cells. CCK‐8 assay was carried out to determine the I_C50_ values and above inhibition rates. Data were presented as mean ± SD; at least three independent experiments were carried out for the results. Statistical difference was determined through ANOVA, *P < .05, **P < .01 in comparison to control (0 μg/mL DHL control)

Dehydrocostus lactone was purified by repeated column chromatographies from the petroleum ether extract of *Saussureacostus*. The chemical structure and HPLC chromatogram of DHL are shown in Figure 1A,B. DHL was primarily tested to assess the growth inhibition percentage on Hep‐2, TU212 and HBE cell lines through CCK‐8 assay. DHL was potent to inhibit Hep‐2 and TU212 cell proliferation while being less toxic to the human larynx epithelial cell line HBE cells (IC_50_ > 25 μg/mL),as presented in Figure 1C,D. After 6, 12, and 24 hours of incubation, cytotoxicity and viability of Hep‐2 and TU212 cells were tested and shown in Figure 1E‐I. From the results, it was deduced that DHL suppressed cell viability on Hep‐2 and TU212 cells in dose‐ and time‐dependent manners. Photomicrographs showed that DHL treatment induced Hep‐2 and TU212 cell shrinkage, distortion and disengage of cellular matrix without obvious impact on HBE cells, and it was verified that DHL has a low toxic effect on normal human laryngeal epithelial cells (Figure 1J). Cell proliferation inhibition is affected by cell cycle arrest.^21^ Therefore, we further evaluated whether DHL affects cell cycle arrest. Western blot analysis showed that typical anti‐proliferative proteins p53 and p21 expression were enhanced, accompanied by the decrease of cyclin D1 in Hep‐2 and TU212 cells after DHL treatment (Figure 1K‐M). These results suggested that DHL has an inhibitory effect on Hep‐2 and TU212 cell proliferation, and may induce apoptosis by regulating the expression of apoptotic pathway and survival pathway members.

### 3.2 DHL suppressed the migratory ability of laryngeal carcinoma cells

**Figure 2:**
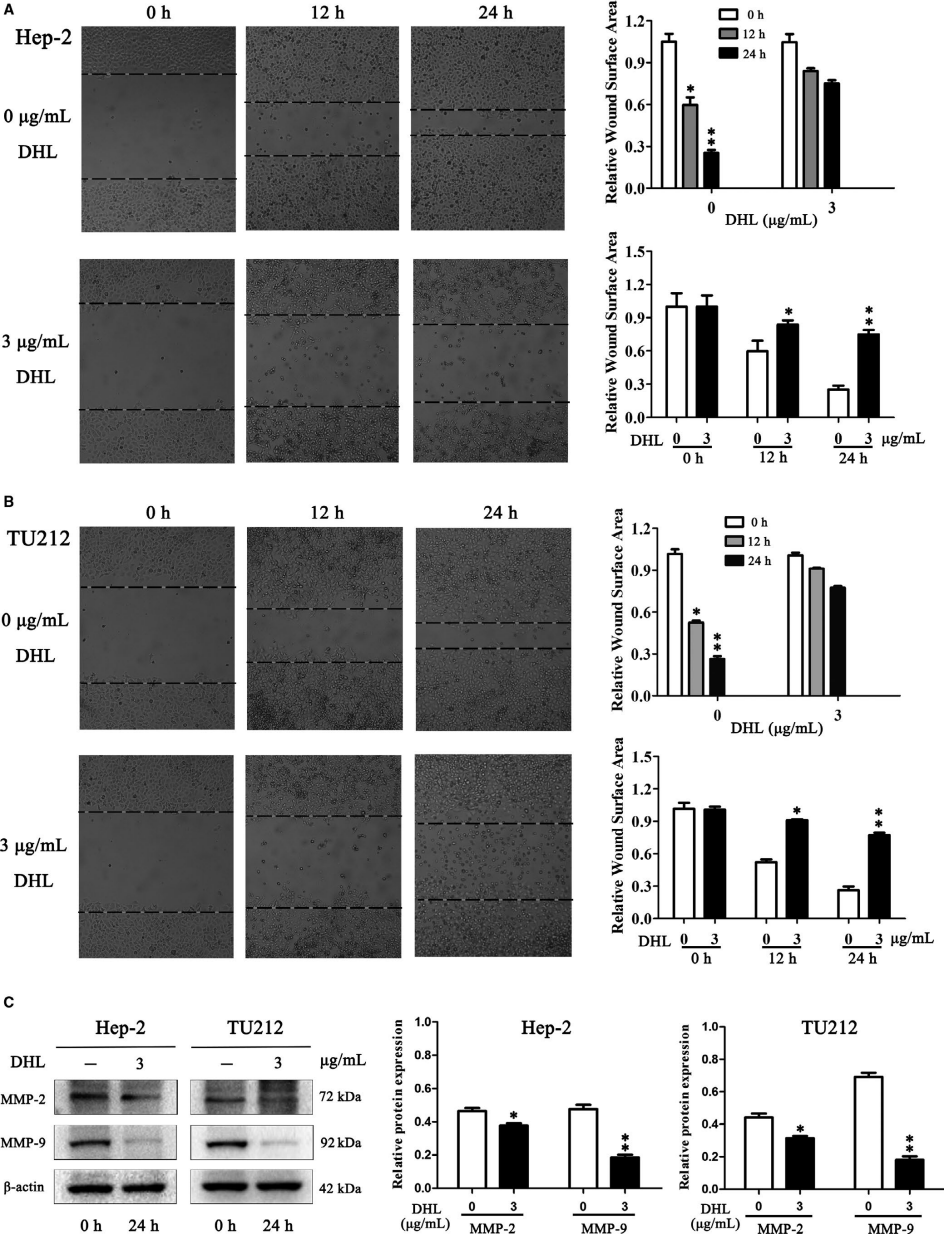
Dehydrocostus lactone (DHL) treatment inhibited laryngeal carcinoma cell migration. A and B, Assay for wound healing was carried out on Hep‐2 and TU212 cells. The cells were treated with 0, 3 µg/mL of DHL and photomicrography was done at 0, 12 and 24 h. C, Relative levels of MMP‐2 and MMP‐9 in Hep‐2 and TU212 cells. Data are presented as mean ± SD in three experiments done independently. ANOVA was used to analyse the statistical difference, *P < .05, **P < .01 in comparison to control (0 μg/mL of DHL was the control)

The assay for wound healing was employed to detect the effect of DHL on the migratory ability of laryngeal carcinoma cells. As shown in Figure 2A,B, treatment with DHL significantly inhibited the migration of laryngeal carcinoma cells. Matrix metalloproteinases (MMPs) play a vital role in tumour migration and invasion by degradation of extracellular matrix.^22^ Hence, we assessed the expression of these key proteins associated with cell migration and invasion, including MMP‐9 and MMP‐2, and found that DHL significantly impeded the expressions of MMP‐2 and MMP‐9 (Figure 2C). Therefore, DHL strongly inhibited the migration of laryngeal carcinoma cells.

### 3.3 DHL induces laryngeal carcinoma cell apoptosis and inhibits the tumour growth

**Figure 3:**
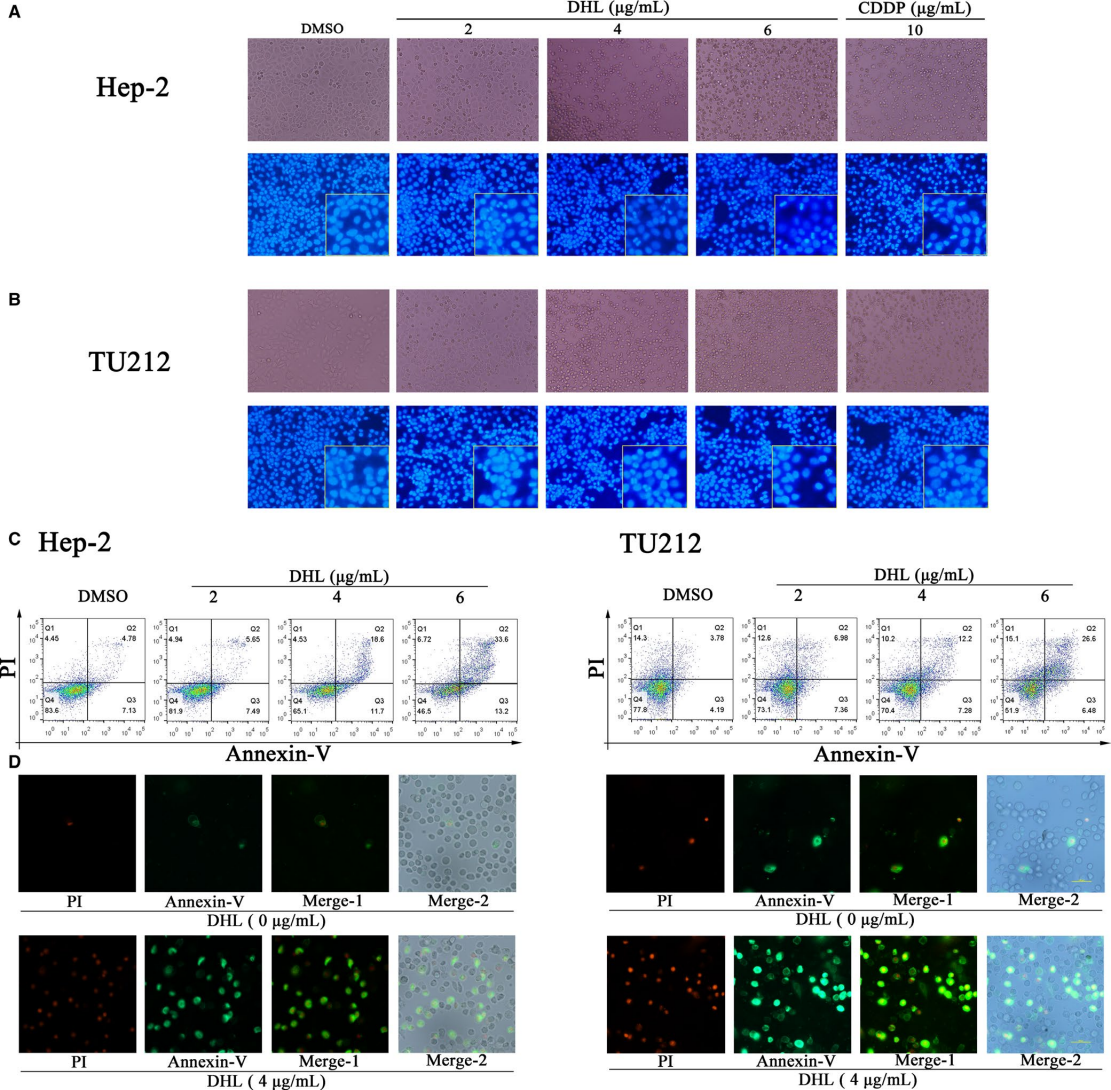
Dehydrocostus lactone (DHL) induced apoptosis of laryngeal carcinoma cells in vitro. A and B, Hep‐2 and TU212 cells were treated for 12 h with 0, 2, 4 and 6 μg/mL of DHL and 10 μg/mL of CDDP, after which the cells were observed and photographed under a phase contrast microscope; fixation of Hep‐2 and TU212 cells followed by incubation with Hoechst 33258 was done for 30 min, after which cells were observed for morphological changes under a fluorescence microscope and photographed. The positive control was CDDP group and the apoptosis characteristics of cell shape are indicated by white points. C and E, Hep‐2 and TU212 cells were treated for 12 h with 0, 2, 4 and 6 μg/mL of DHL. After Annexin V‐FITC/PI staining, assessment of apoptosis was flow cytometrically assessed. Above fractions were calculated by FlowJo X. D and F, Annexin V‐FITC/PI was performed on Hep‐2 and TU212 cells after 12 h treatment with DHL, and apoptosis was observed by fluorescence microscope. Two‐colour filter was used to observe under fluorescence microscope. Annexin V‐FITC fluorescence was green, and PI fluorescence was red

Apoptosis is a molecular mechanism of cell death, and the induction of apoptosis is the key mechanism of anti‐cancer therapy.^23^ Therefore, we investigated the correlation between DHL‐induced growth inhibition of laryngeal carcinoma cells and increased apoptosis of laryngeal carcinoma cells. After 12 hours incubation of DHL with laryngeal carcinoma cells, we evaluated the morphology of laryngeal cancer cells with and without DHL treatment. Most of the laryngeal carcinoma cells treated by DHL showed a trend of gradual shrinking necrosis. As Hoechst 33258 staining shown, the identical apoptosis characteristics triggered by CDDP (chromatin condensation and apoptotic body) were also progressively observed in DHL‐treated laryngeal carcinoma cells (Figure 3A,B). FACS (PI and Annexin V) analysis also showed that DHL treatment resulted in an increase of apoptotic population (Q2 and Q3). The overall rate of apoptosis of Hep‐2 cells was 13.14%, 30.30% and 46.80%, respectively, at DHL concentrations of 2, 4 and 6 μg/mL (Figure 3C). Likewise, in the TU212 cells, the percentage of cells undergoing apoptosis was 14.34%, 19.48% and 33.08% after the treatment with DHL (Figure 3E). Fluorescence microscopy showed that DHL significantly induced apoptosis of Hep‐2 and TU212 cells (Figure 3D,F). These results indicated that DHL inhibited the growth of Hep‐2 and TU212 cells by inducing apoptosis according to the dose administered.

**Figure 4:**
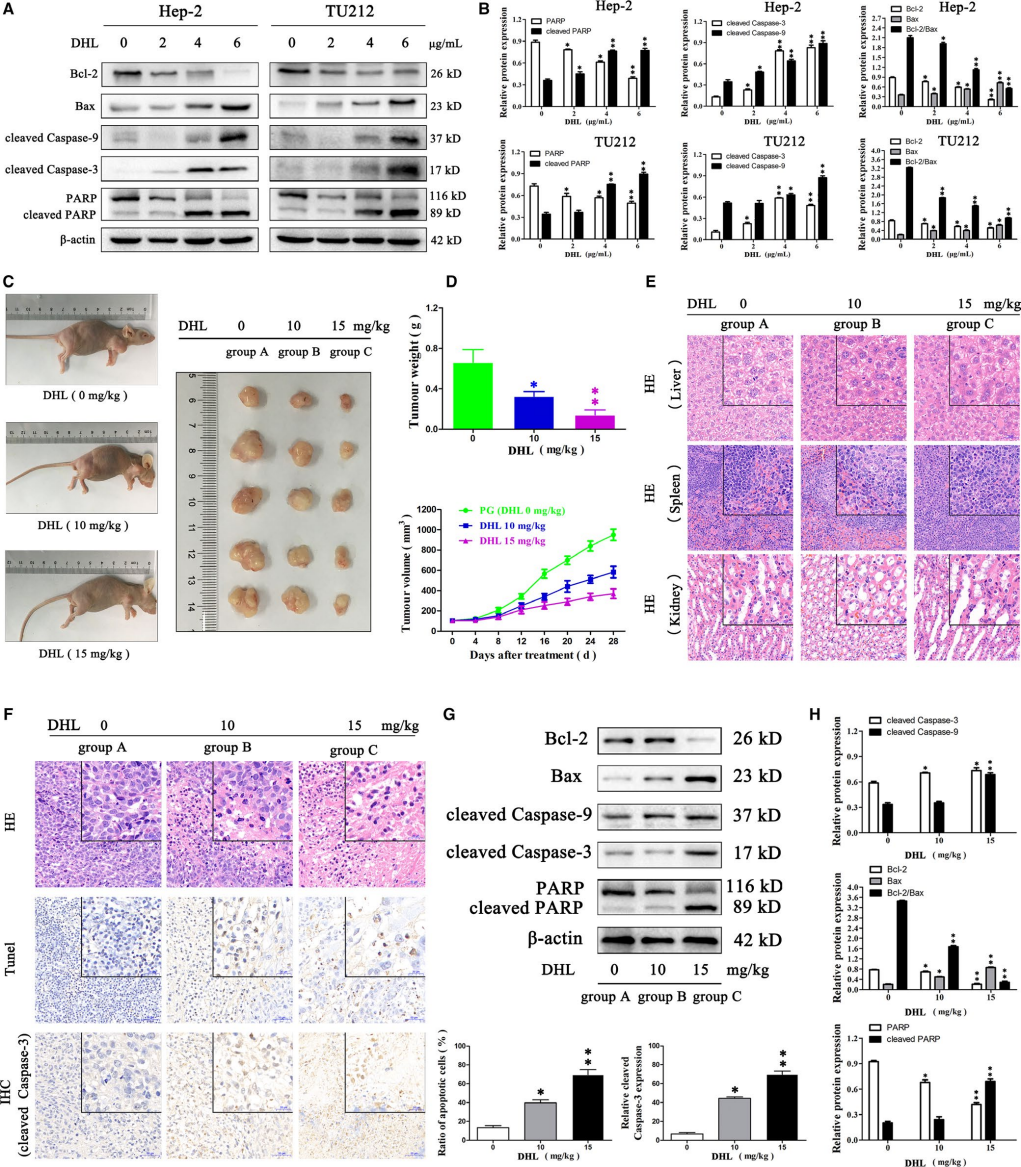
Dehydrocostus lactone (DHL) induced apoptosis of laryngeal carcinoma cells, and pro‐apoptotic and anti‐proliferation effect of DHL in Hep‐2 bearing nude mice model. A, Hep‐2 and TU212 cells were given a treatment with 0, 2, 4 and 6 µg/mL of DHL for 12 h, following which processing of whole‐cells lysates was done for Western blotting and probed with the specified antibodies. The effects of DHL on mitochondrial apoptotic proteins were analysed by Western blot. B, Relative levels of PARP, cleaved PARP, cleaved caspase‐3, cleaved caspase‐9, Bax and Bcl‐2 and ratio of Bcl‐2/Bax proteins in Hep‐2 and TU212 cells. C, Inhibitory effect of DHL administration on the proliferation of Hep‐2 xenograft tumour model in nude mice. D, When tumour volume reached 100 mm^3^, DHL (10 and 15 mg/kg) or PG (5% V/V) was given every 2 d for 4 wk; nude mouse was killed and dissection of xenografts was done to measure weights; after implantation, every 4 d the volume of tumour was measured after implantation. E, HE staining sections of the spleen, liver and kidney of tumour‐bearing mice showed no significant difference among the groups. F, Tumour tissue sections of Hep‐2 bearing nude mice were subjected to HE staining, Tunnel staining and IHC. HE‐stained tumour tissue sections were used for histological morphology. Quantification of apoptotic cells was determined by Tunnel staining. Expression of cleaved caspase‐3 was visualized and quantified by IHC. G, The effects of DHL on mitochondrial apoptotic proteins were analysed by Western blot in xenografts lysates. H, DHL treatment promoted the cleavage of caspase‐3, 9 and PARP, and reduced the proportion of Bcl‐2/Bax in vivo. Data are presented as mean ± SD in three experiments done independently. ANOVA was used to analyse statistical difference, *P < .05, **P < .01 in comparison to control (0 µg/mL DHL was the control in vitro and 0 mg/kg DHL was the control in vivo)

Further, to detect the expression of key proteins associated with apoptosis (Bax, Bcl‐2, caspase‐3, caspase‐9 and PARP), Western blotting was carried out. Bax, which is pro‐apoptotic and the anti‐apoptotic Bcl‐2, regulates intrinsic apoptosis senses of death signals.^24^ Previous studies have reported that the balance establishes the onset of apoptosis in mitochondria, and a high Bax/Bcl‐2 ratio is thought to promote apoptosis.^25^ Caspases‐3 and ‐9 act as executioners in apoptosis, and once they are activated, cleavage of their precursor forms occurs.^26^ PARP is the target protein of caspase‐3, affecting the repair of cancer cells. Activated caspase‐3 cleaves PARP and prevents DNA from repairing itself.^27^ DHL treatment significantly enhanced the expression of cleaved caspase‐3 and ‐9, cleaved PARP and dose‐dependently reduced the ratio of Bcl‐2/Bax (Figure 4A,B). Taken together, these results suggested that DHL can potentially induce apoptosis in cancer cells of the larynx by activating the apoptotic pathway of the mitochondria.

Bearing nude mouse model carrying Hep‐2 was used to assess the anti‐tumour effects of DHL against laryngeal carcinoma cells in vivo. Treatment with DHL resulted in inhibition of Hep‐2 xenografts growth in mice and led to reduction in tumour weights based on the doses administered (Figure 4C,D). Tissue sections of the spleen, liver and kidney showed no significant difference among groups A, B and C, indicating that DHL was less toxic to nude mice (Figure 4E). Considering the noticeable pro‐apoptotic action of DHL against laryngeal carcinoma cells in vitro, we determined whether DHL induced Hep‐2 cells apoptosis in vivo. Xenografts were collected from Hep‐2 bearing nude mice and visualized by HE, Tunnel staining and IHC. HE staining results showed that DHL treatment caused tumour chromatin agglutination and loose structure. DHL dose‐dependently enhanced the apoptotic nuclei of the xenograft sections after Tunnel staining analysis. Similar results were observed in IHC staining. DHL‐treated (groups B and C) xenograft samples exhibited high cleaved caspase‐3 staining while PG‐treated (group A) tumour samples were not obvious (Figure 4F). Western blot analysis proved the observation in IHC staining and suggested that DHL‐induced apoptosis also resulted from the mitochondrial apoptotic pathway (Figure 4G,H). Thus, treatment with DHL impeded the growth of tumour and induced laryngeal carcinoma cell apoptosis through the apoptotic pathway of the mitochondria, which further confirmed that DHL has potential medicinal value in the anti‐laryngeal cancer treatment.

### 3.4 DHL induces apoptosis of laryngeal carcinoma cells through endoplasmic reticulum stress (ERS) pathway

**Figure 5:**
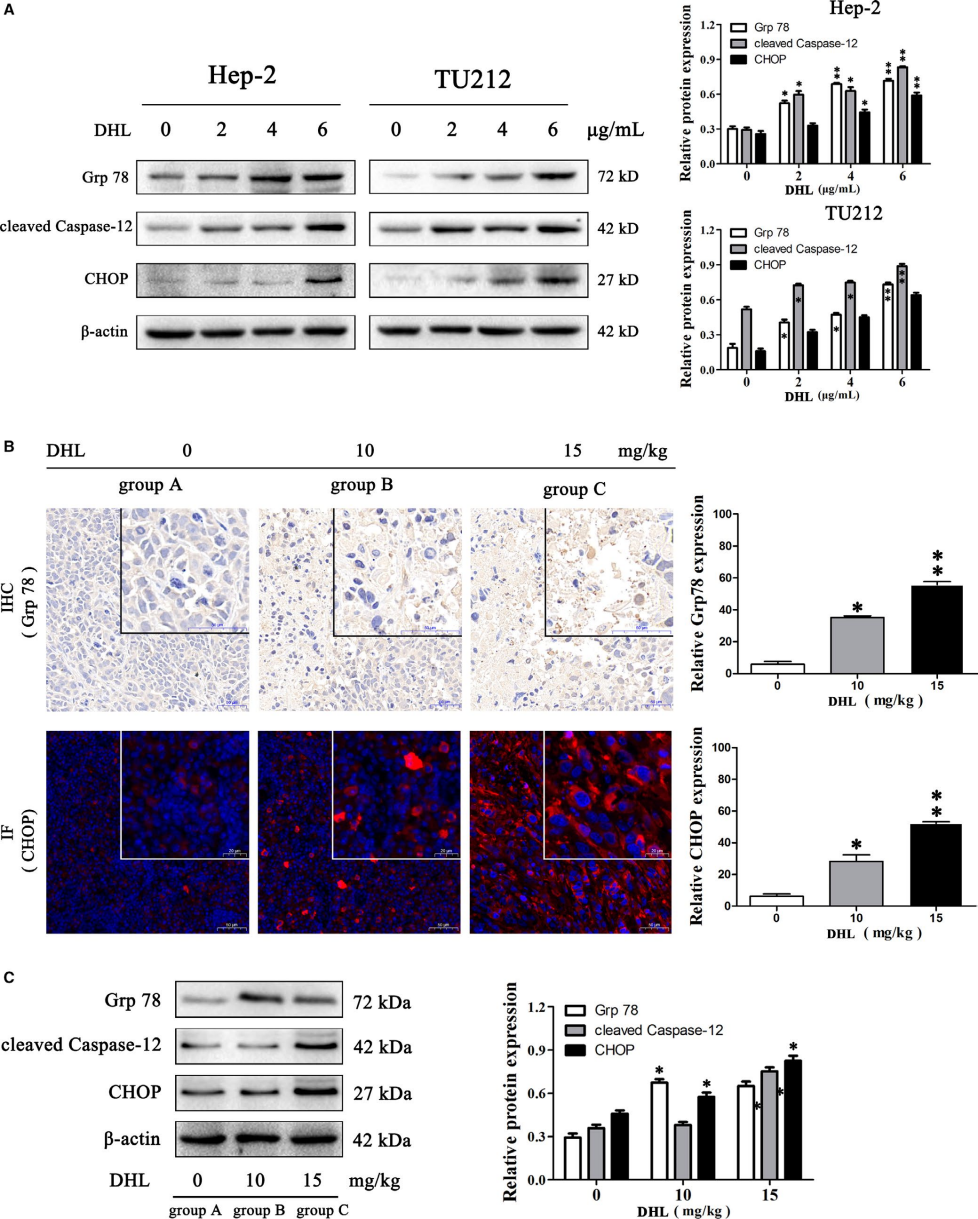
Dehydrocostus lactone (DHL) induces apoptosis in cancer of the larynx through ERS signalling pathway. A, Hep‐2 and TU212 cells were treated for 12 h with 0, 2, 4 and 6 µg/mL of DHL, following which processing of whole‐cell lysates was done for analysis through Western blotting and probed with the specified antibodies. The effects of DHL on ERS pathway proteins were analysed by Western blot. B, Tumour tissue sections of Hep‐2 bearing nude mice were prepared and subjected to IHC and IF. Expression of Grp 78 was visualized and quantified by IHC. Expression of CHOP was visualized and quantified by IF. DHL treatment increased the expression of Grp 78 and CHOP in laryngeal squamous carcinoma tissues. C, The effects of DHL on ERS pathway proteins were analysed by Western blotting in xenografts lysates. DHL treatment in vivo promoted Grp 78, cleaved caspase‐12 and CHOP. Data are presented as mean ± SD in three experiments done independently. ANOVA was used to analyse statistical difference, *P < .05, **P < .01 in comparison to control

The endoplasmic reticulum is an organelle closely related to protein folding and assembly, lipid synthesis and free calcium storage.^28^ Responding to ER stress, cells activate a series of steady‐state responses, collectively referred to as UPR (unfolded protein response), to maintain the homeostasis of the endoplasmic reticulum.^29^ One major pathway of the UPR is the up‐regulation of ER chaperones, such as the glucose‐regulated protein 78(GRP78), which can be involved in the repair of unfolded proteins.^30^ Moderate ERS can reduce cellular dysfunction and increase the possibility of survival, whereas excessive and prolonged ERS leads to apoptosis. ERS‐induced apoptosis is mediated through transcription‐induced C/EBP homologous protein (CHOP), which activates the caspase‐12‐dependent pathway.^31^, ^32^ GRP78 is a central regulator of endoplasmic reticulum function, and an increase in GRP78 expression suggests endoplasmic reticulum stress. The induction of GRP78 has been widely used as a marker for the occurrence of UPR and ER stress. Caspase‐12 is an ER in situ caspase that has been suggested as a crucial moderator of apoptosis induced by ER stress. Caspase‐12 is specifically cleaved and activated during ER stress, but not bymitochondrial‐mediated apoptotic signals.^33^, ^34^ Similarly, CHOP expression levels are elevated during ER stress progress and may mediate endoplasmic reticulum stress‐related apoptosis.^35^ Hence, we carried out Western blot to assess the expression of cleaved caspase‐12, Grp78 and CHOP, which was seen to significantly enhance the expression of Grp78, cleaved caspase‐12 and CHOP depending on the dose (Figure 5A). The results showed that endoplasmic reticulum stress was also a part of DHL‐induced apoptosis of laryngeal carcinoma cells. The action of DHL on ERS‐induced apoptosis was further confirmed by analysing the above xenografts proteins. As anticipated, DHL treatment increased the expressions of Grp78, cleaved caspase‐12 and CHOP in vivo. Immunohistochemistry and immunofluorescence staining further confirmed that DHL promoted Grp78 and CHOP expressions in xenograft tissues (Figure 5B,C). Further, these results suggested that apoptosis induced by DHL was also carried out by the apoptotic signalling with ERS.

### 3.5 DHL inhibited Akt activity in laryngeal carcinoma

**Figure 6:**
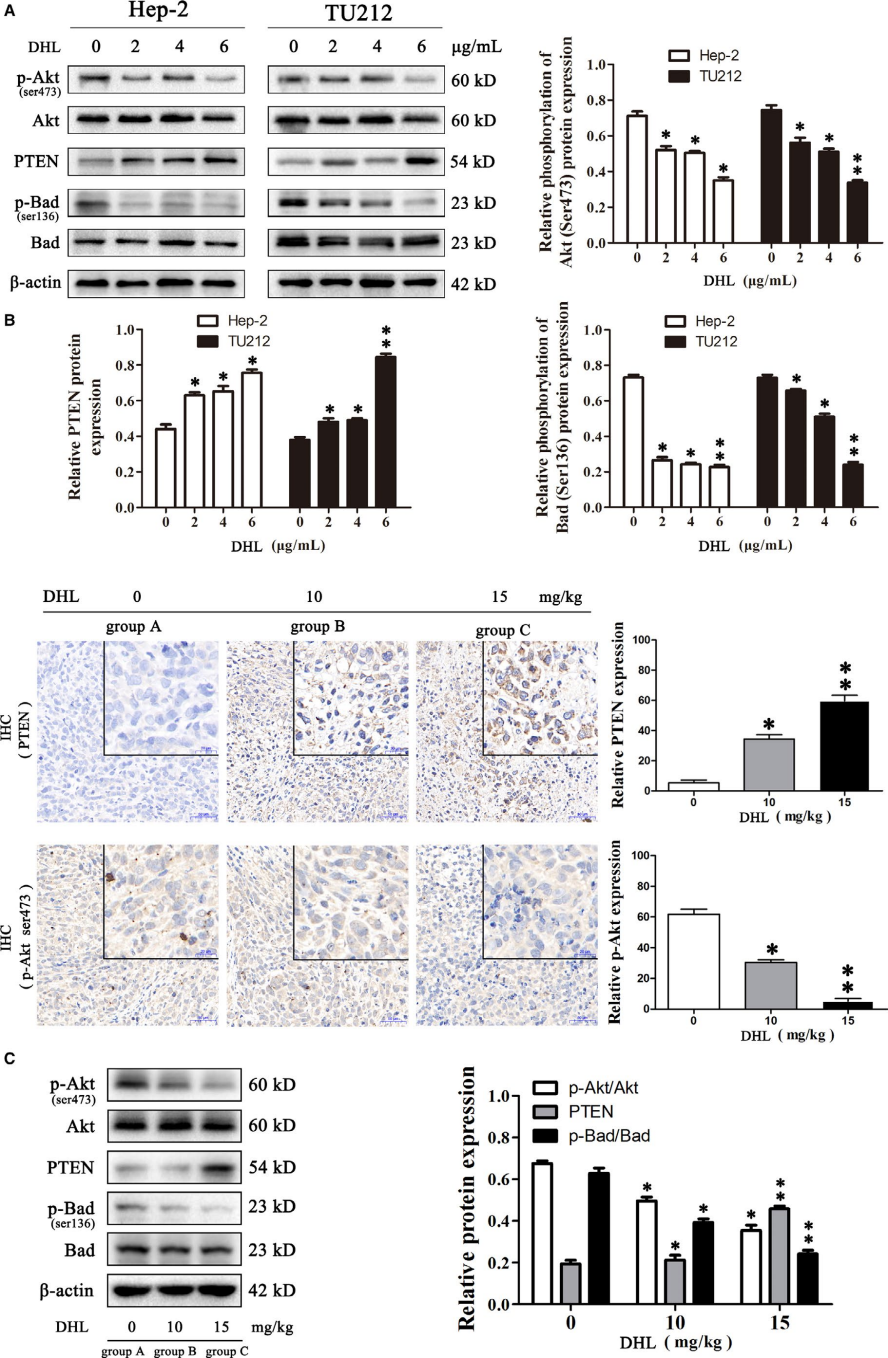
Dehydrocostus lactone (DHL) induces laryngeal carcinoma cells apoptosis through PTEN/Akt/Bad pathway. A, Hep‐2 cells and TU212 cells were treated for 12 h with 0, 2, 4 and 6 µg/mL of DHL, following which processing of whole‐cell lysates was done for analysis through Western blotting and probed with the specified antibodies. The effects of DHL on PTEN/Akt/Badpathway proteins were analysed by Western blot. B, Tumour tissue sections of Hep‐2 bearing nude mice were subjected to IHC. Expression of PTEN and p‐Akt was visualized and quantified by IHC. C, The effects of DHL on PTEN/Akt/Bad pathway proteins were analysed by Western blot in xenografts lysates. Data are presented as mean ± SD in three experiments done independently. ANOVA was used to analyse statistical difference, *P < .05, **P < .01 in comparison to control

**Figure 7:**
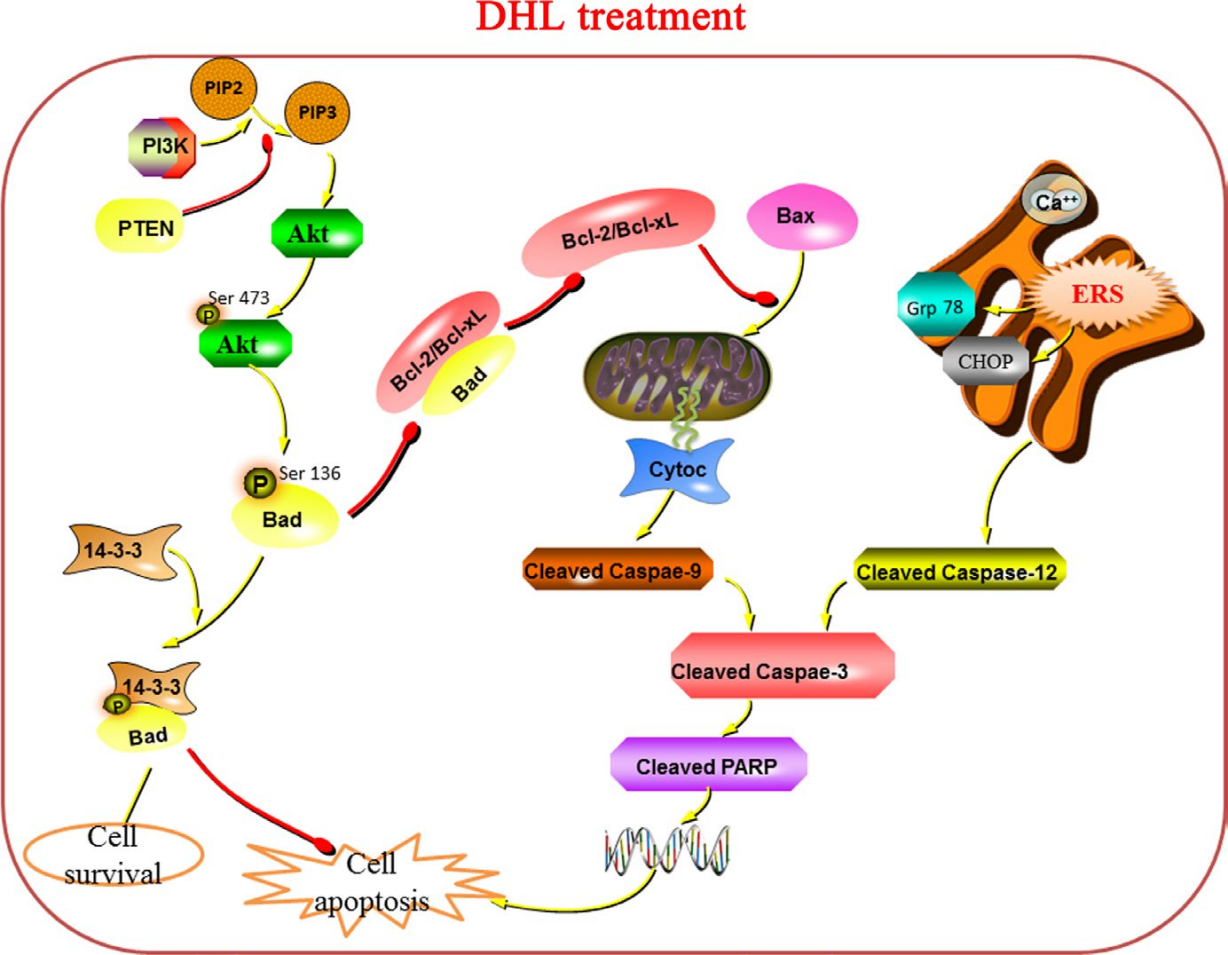
Schematic to represent the proposed molecular events in DHL‐induced apoptosis in laryngeal carcinoma cells

Akt is a vital regulator in PI3K/Akt/Bad signalling pathway suppressing cell apoptosis, which frequently become hyperphosphorylation for activation in cancer cells. Expression and function of PTEN in human malignant tumour tissues and tumour cell lines can exert tumour inhibition through a variety of ways, the most important of which is to inhibit the PI3K/Akt pathway, in which PTEN has the role of ‘molecular switch’.^36^, ^37^ Bad protein is a typical Akt‐phosphorylated protein involved in cellular proliferation and apoptosis.^38^ Therefore, we were interested in the involvement of DHL in Akt signalling. Western blot analysis revealed that DHL treatment dose‐dependently increased the expression of PTEN and down‐regulated the phosphorylation level of Akt accompanied by decreasing the phosphorylation of Bad (Figure 6A). We further measured phosphorylation levels of Akt in xenograft tissues. Similarly, DHL administration inhibited Akt activity and correspondingly resulted in the dephosphorylation of its downstream Bad in tumour tissues of nude mice. Immunohistochemical analysis results showed that DHL increased the expression of PTEN and decreased the expression of p‐Akt, further confirming the inhibitory effect of DHL on Akt in vivo (Figure 6B,C)*.* These results suggested that DHL is capable of inhibiting Akt activity, which appeared to implicate in DHL‐induced laryngeal carcinoma cell apoptosis. Taken together, our data strongly suggested that DHL‐induced apoptosis on laryngeal carcinoma cells is also regulated by PI3K/Akt/Bad signal pathway. The molecular signalling mechanism of DHL against laryngeal carcinoma cells is shown in Figure 7.

## 4 DISCUSSION

In our study, dehydrocostus lactone (DHL), a natural sesquiterpene, was isolated from the roots of *Saussurea costus*. Induction of apoptosis by DHL has been reported in many types of cancer cells.^7^, ^8^, ^9^, ^10^ However, there are few reports on the cytotoxic activity of DHL in laryngeal carcinoma cells, and the molecular mechanism of DHL inducing apoptosis of laryngeal carcinoma cells is still unclear. To determine whether DHL has the activity of inhibiting laryngeal carcinoma, we tested its cytotoxic activity and anti‐proliferative effect on an array of laryngeal carcinoma cell lines in vitro. Our study found that DHL inhibited laryngeal carcinoma cell proliferation, depending on dose and time, without inducing cytotoxicity in laryngeal epithelial cells. Therefore, we carried out several in vivo and in vitro experiments to further determine the role of DHL in anti‐laryngeal carcinoma. In vitro, it was shown that anti‐laryngeal cancer effects of DHL were time‐dependent, so we speculated whether DHL’s anti‐laryngeal carcinoma activity was related to inhibiting cell proliferation, and Western blot analysis verified our hypothesis. DHL delayed cell proliferation by blocking the cell cycle of laryngeal carcinoma. Invasion and metastasis are the important characteristics of many squamous carcinomas, so inhibiting the invasion and migration of laryngeal carcinoma is also one of the focuses of our study. MMP‐2 and MMP‐9 gelatinases promote invasion and migration by degrading collagen and fibronectin matrices.^39^ Thus, the expression levels of matrix metalloproteinases (MMPs), including MMP‐9 and MMP‐2, are used as a hallmark for tumour migration and invasion.^22^ Through experimental analysis, it is concluded that low concentration of DHL can inhibit the migration of laryngeal carcinoma cells. Apoptosis is a self‐destructive, intrinsic process involved in the treatment of many cancers. Inadequate apoptosis can disrupt the sensitive equilibrium of cellular processes and eventually lead to carcinogenesis.^40^ In the actual drug development, various targeted drugs developed in the exhaustive biochemical mechanism of understanding of apoptosis kill cancer cells by inhibiting the key molecules in the apoptosis signalling pathway mutations or increasing the activation of the conservative apoptosis mechanism.^41^

The mitochondria have been shown a crucial role in apoptosis.^42^, ^43^ The relative Bcl‐2 and Bax expressions and caspase‐9 and caspase‐3 activation are key factors in the signalling pathways mediating mitochondrial apoptosis, in which Bax and Bcl‐2 regulate permeability in mitochondria. The change in ratio of Bax/Bcl‐2 causes the cytochrome C release, which is related to the activation of caspases‐3 and ‐9, and finally leads to apoptosis. Morphological observation and FACS analysis of laryngeal carcinoma cells treated with different concentrations of DHL showed that DHL could promote the apoptosis of laryngeal carcinoma cells. Based on the good in vitro pro‐apoptotic activity of DHL on laryngeal cancer cells, nude mouse model carrying Hep‐2 cell line was used to assess the in vivo anti‐tumour effects of DHL against laryngeal carcinoma. DHL obviously inhibited the growth of transplanted tumours. As per the results of Western blot, in vitro and in vivo samples exhibited reduced Bcl‐2/Bax ratio in a dose‐dependent manner after DHL treatment, while the expressions of caspase‐9, caspase‐3 and cleaved PARP were increased dose‐dependently. These data suggest that DHL may be a potential anti‐laryngeal cancer agent by activating the apoptosis signalling pathway of the mitochondria. Studies have also recently pointed out that ERS‐mediated apoptosis signalling pathway is a new apoptosis mechanism. Therefore, we made a hypothesis that ERS‐mediated apoptosis was also involved in DHL‐induced apoptosis of laryngeal carcinoma cells. In vitro, the results showed that DHL treatment significantly enhanced the Grp78, cleaved caspase‐12 and CHOP expressions in a dose‐dependent manner. The results are consistent with those of our in vivo experiments, which confirm our previous hypothesis. The in vivo and in vitro results showed that DHL induced apoptosis of laryngeal carcinoma cells through ER apoptosis signalling pathway.

Malignant transformation of tumour is caused by oncogene and tumour suppressor gene mutation.^44^ Abnormally rich transformed cells often show increased cellular stress and eventually led to death. As a result, cancer cells often rely on survival signals that do not normally play such an important role in normal cells. Therefore, targeting these signals can reactivate the death process and increase sensitivity to tumour cells.^45^ DHL had a good effect on pro‐apoptosis and anti‐proliferation of laryngeal cancer cells in vitro, and promoted the apoptosis of Hep‐2 xenograft tumour in nude mice by activating mitochondrial apoptosis and endoplasmic reticulum stress pathways. Based on these apoptotic pathways, we have been suggested that it was related to a survival signal. Interestingly, we found that phosphorylation of Akt in laryngeal cancer cells decreased after DHL treatment. This also confirmed that DHL can stimulate apoptosis of laryngeal cancer cells by affecting survival signals. Normally, apoptosis and proliferation are tightly regulated through highly coordinated signalling pathways. Akt is one of the key integrators regulating survival and proliferation, and its phosphorylation at Ser473 is necessary for the activation of Akt, while tumour cells often benefit from Akt hyperphosphorylation to resist cell stress and apoptosis. Akt actively regulates proliferation by degrading cyclin D1 and inhibits p53 by activating MDM2.^38^, ^46^ Activated Akt inhibits apoptosis by phosphorylation and inactivation of several pro‐apoptotic proteins, including Bad and caspase‐9, which are closely related to mitochondrial apoptosis. Moreover, apoptotic proprotein Bad can be deactivated by Ser112 or Ser136 phosphorylation. Particularly, Akt phosphorylates it at Ser136,^47^ after which, Bad attaches 14‐3‐3 protein subtypes and remains isolated in the cytoplasm, unknot able to bind Bcl‐2 or Bcl‐xL.^48^ Thus, Bad is deemed to be the intersection of pro‐apoptotic and anti‐apoptotic regulatory cascades, and the Akt/PI3K pathway is directly linked to the apoptotic mechanism.^49^ We observed that DHL treatment down‐regulated the phosphorylation level of Akt accompanied by decreasing the phosphorylation of Bad in laryngeal carcinoma in vivo as well as in vitro. Our results suggest that Akt dephosphorylation Bad is more likely to form a heterodimer with Bcl‐xL/Bcl‐2, deactivating it and enabling Bax to trigger mitochondrial apoptosis.

In conclusion, our study revealed that DHL‐induced apoptosis of laryngeal carcinoma cells activated mitochondrial apoptosis pathway by impeding Akt/PI3K/Bad signalling pathway and stimulated endoplasmic reticulum stress‐mediated apoptosis for the first time. We have clearly explained the related research and molecular mechanism of dehydrocostus lactone against laryngeal cancer in vivo and in vitro in this study. However, we are still aware of the lack of systematic evaluation during the research process. There is a lack of preclinical trials using DHL for multicenter, large‐sample, double‐blind and randomized chemoprevention. Therefore, we will pay close attention to this phase of DHL in future studies.

## CONFLICT OF INTEREST

All authors declare no conflict of interest.

## AUTHORS' CONTRIBUTIONS

RZ, QW and XZY conceived and designed the study. QW and XZY supervised all experiments. RZ, JH, QMW, KWG, CW, WKZ, WXL, QW and XZY performed all experiments. ZR, JH, QW, CW and XZY analysed the data. RZ and XZY wrote the manuscript. All authors read and discussed the manuscript.

## Supporting information

Supplementary Material
