# Supplementary material for: Dehydrocostus lactone inhibits cell proliferation and induces apoptosis by PI3K/Akt/Bad and ERS signalling pathway in human laryngeal carcinoma
Source: J Cell Mol Med. 2020 Apr 21;24(11):6028–42. doi: 10.1111/jcmm.15131 (PMC7294112; doi:10.1111/jcmm.15131)
Supplement: Supplementary file 1 — Supplementary Material [file JCMM-24-6028-s001.docx]

**Dehydrocostus lactone inhibits cell proliferation and induces apoptosis by PI3K/Akt/Bad and ERS signaling pathway in human laryngeal carcinoma**

Ren Zhang^1^, Ji Hao^2^, Qingming Wu^1^, Kaiwen Guo^1^, Chao Wang^1^, Wei Kevin Zhang^2^, Wanxin Liu^1^, Qiang Wang^1^ and Xinzhou Yang^2^

^1^ Institute of Infection, Immunology and Tumor Microenviroment, Hubei Province Key Laboratory of Occupational Hazard Identification and Control, Medical School, Wuhan University of Science and Technology, Wuhan, China;

^2^ School of Pharmaceutical Sciences, South-Central University for Nationalities, Wuhan, China

**Correspondence**

Qiang Wang, Medical College, Wuhan University of Science and Technology, Wuhan 430065, China; Xinzhou Yang, School of Pharmaceutical Sciences, South-Central University for Nationalities, Wuhan 430074, China.

Email: [wangqiang@wust.edu.cn(Q.W.)](mailto:wangqiang@wust.edu.cn(Q.W.)) and [xzyang@mail.scuec.edu.cn](mailto:xzyang@mail.scuec.edu.cn) (XZ.Y.)

**Supplementary Data Contents**

**S1. ^1^ H NMR spectrum of DHL in DMSO**

**S2. ^13^ C NMR spectrum of DHL in DMSO**

**S3. EIMS spectrum of DHL**

**S4.** **Optical Rotation of DHL**

**S5.** **ROESY of DHL**

**S6. Original images of Western blotting (Figure 1)**

**S7. Original images of Western blotting (Figure 2)**

**S8. Original images of Western blotting (Figure 4)**

**S9. Original images of Western blotting (Figure 5)**

**S10. Original images of Western blotting (Figure 6)**


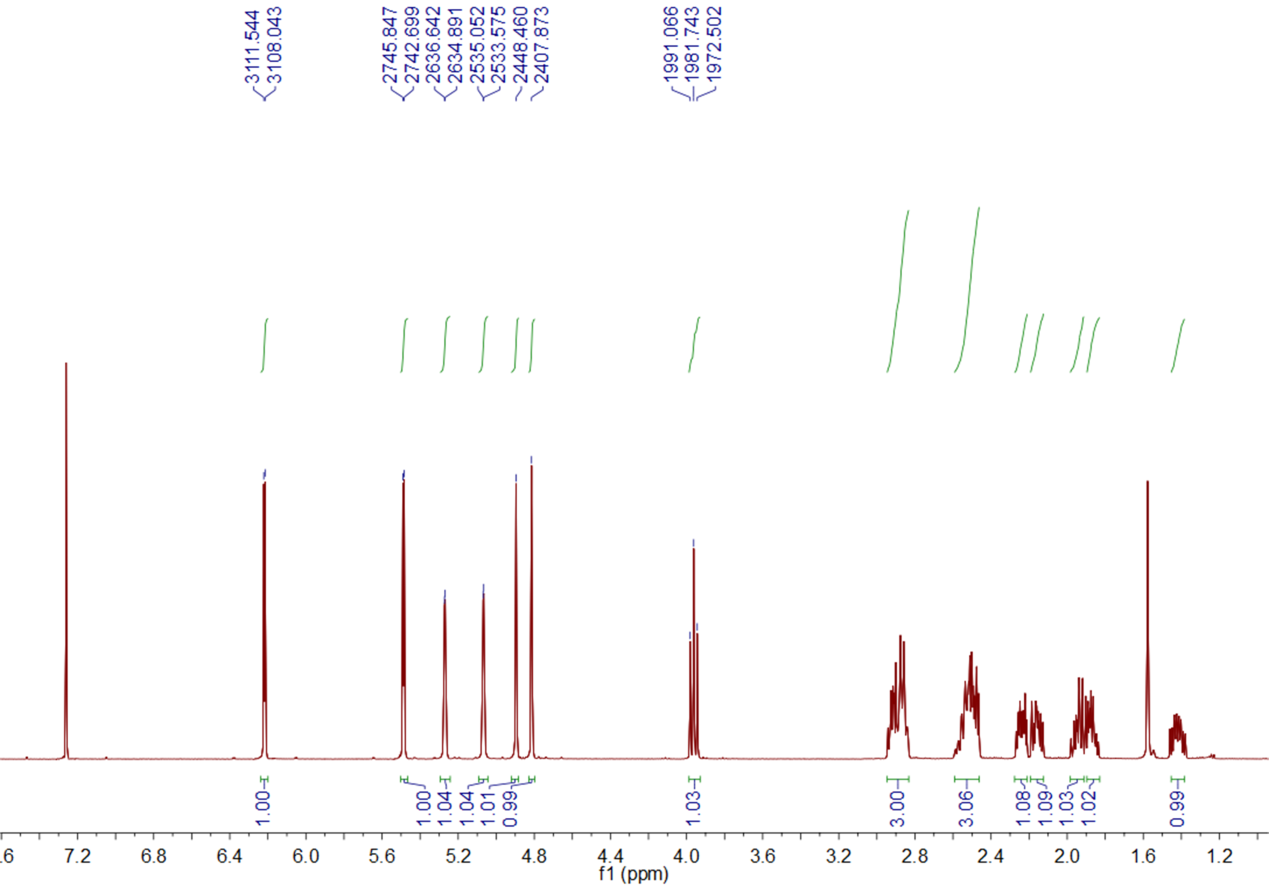


**S1. ^1^H NMR spectrum of DHL in DMSO-d_6_**


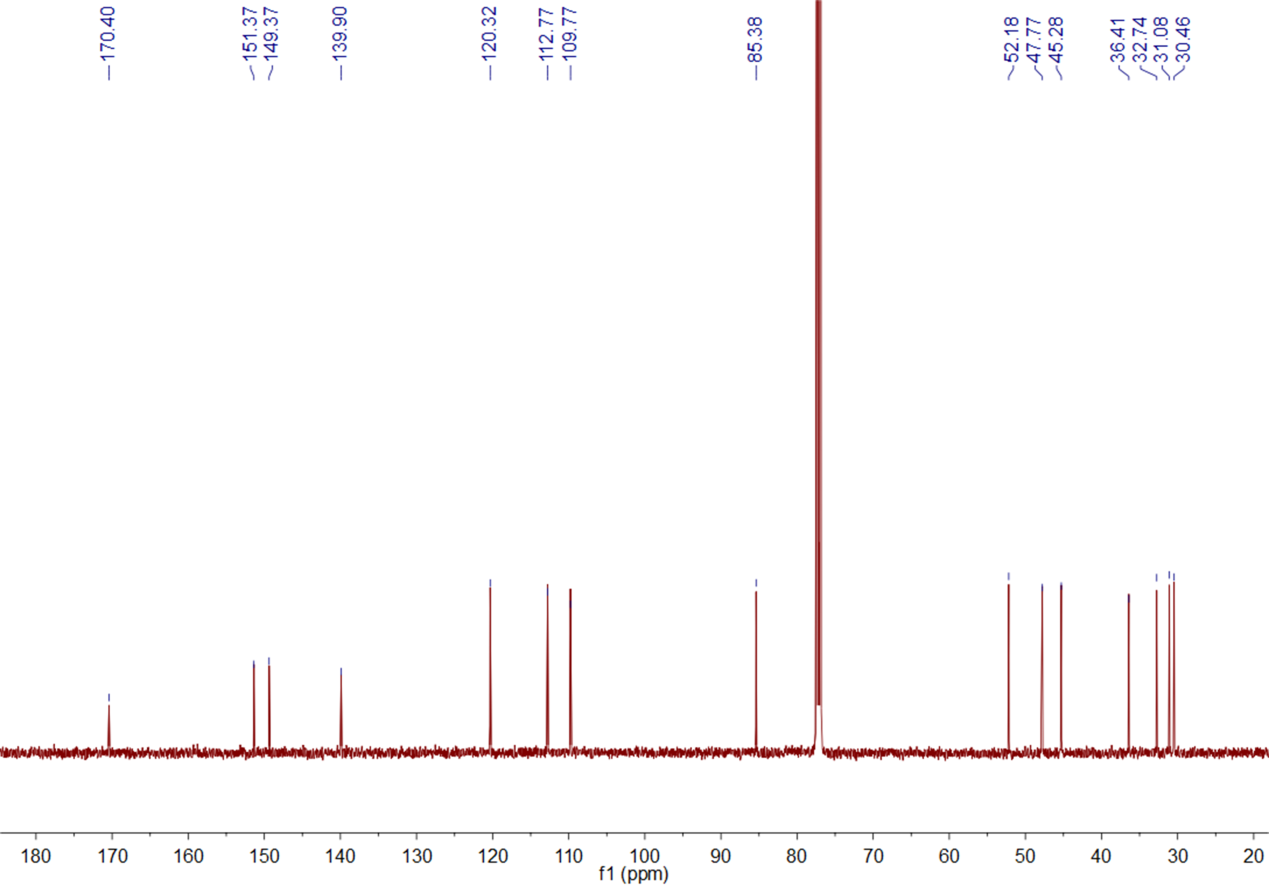


**S2. ^13^C NMR spectrum of DHL in DMSO-d_6_**


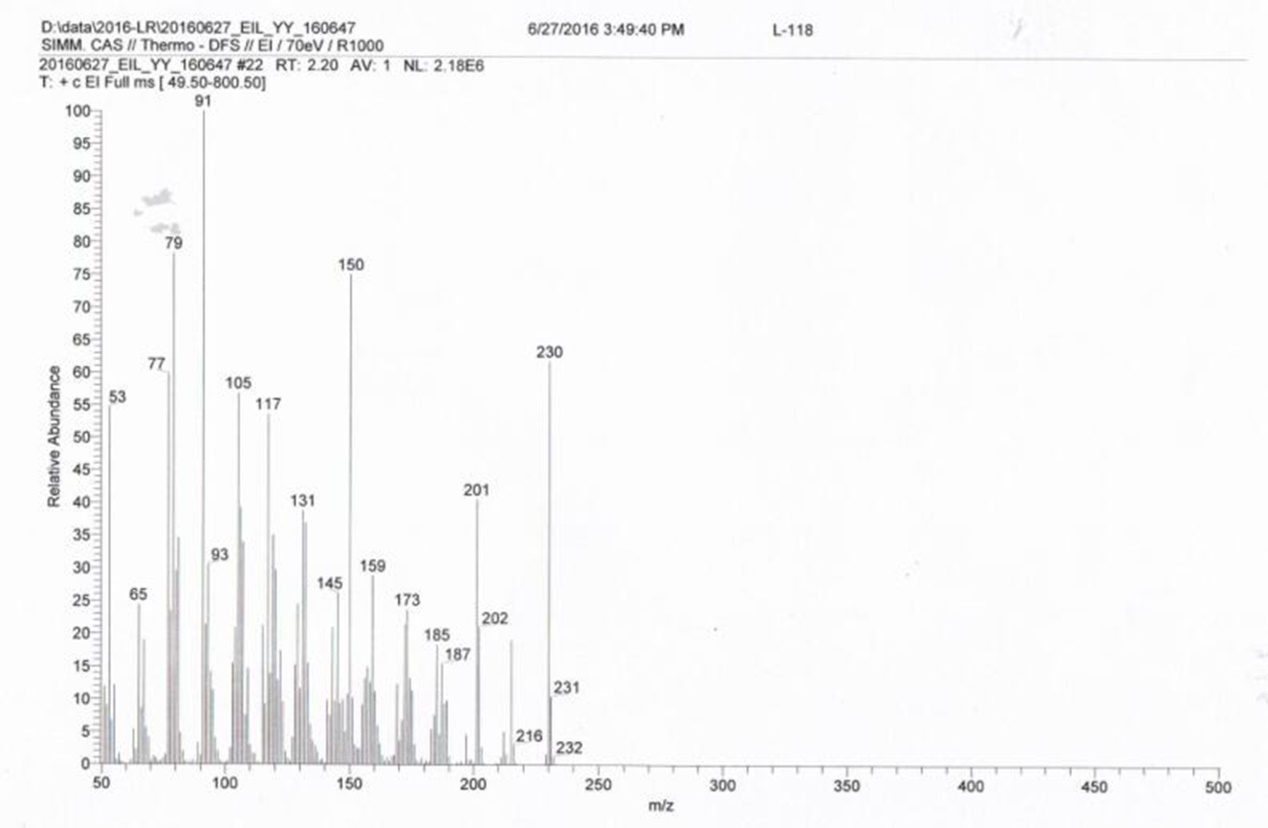


**S3. EIMS spectrum of DHL**


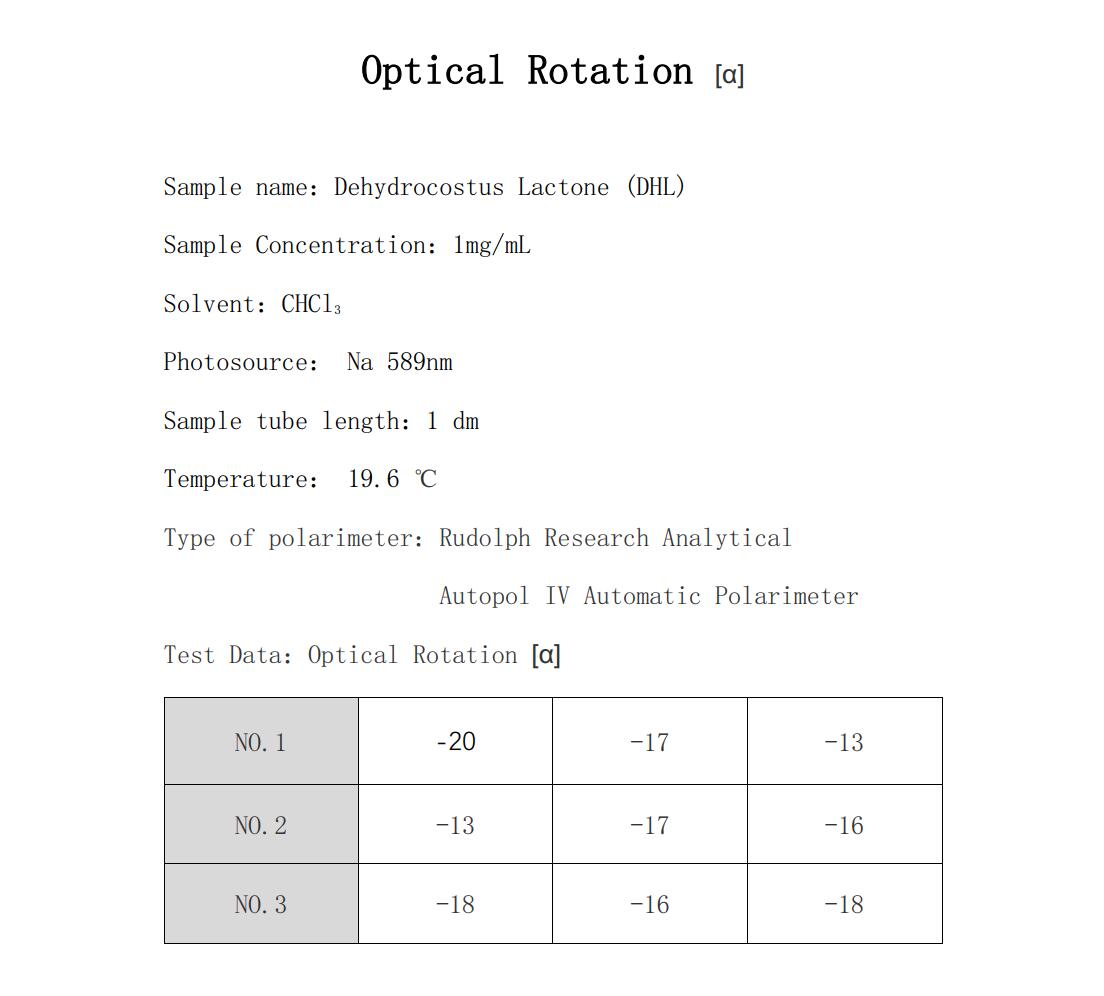


**S4. Optical Rotation of DHL**

**
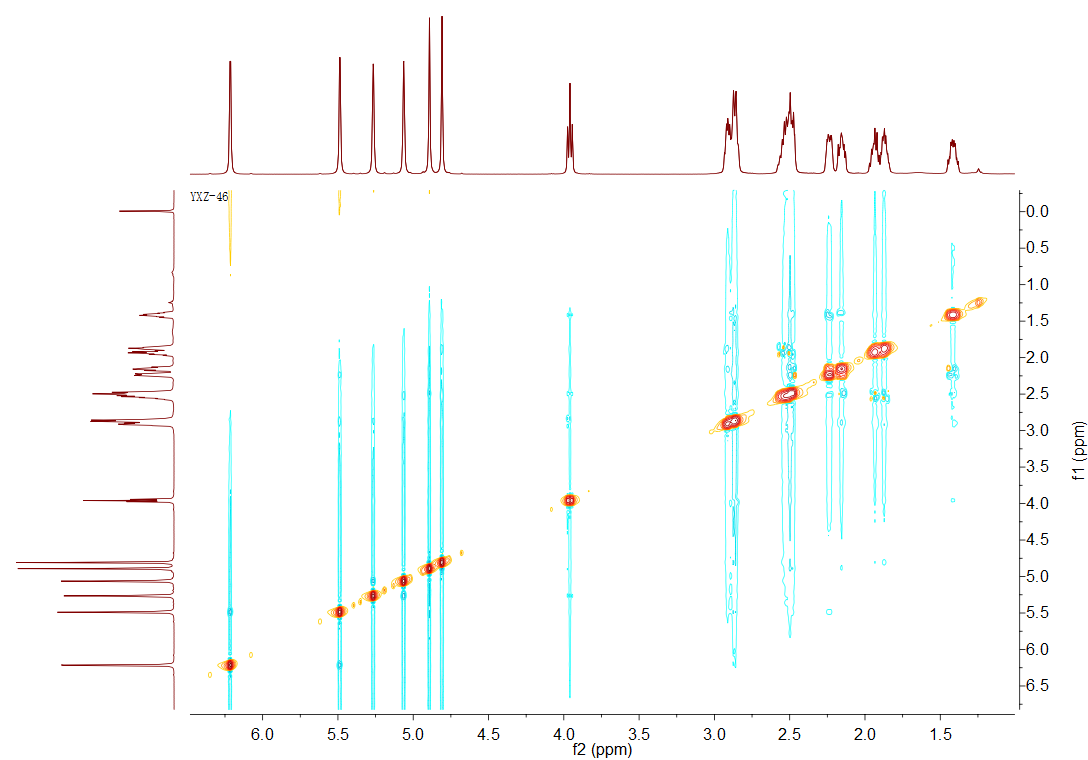
**

**S5. ROESY of DHL**


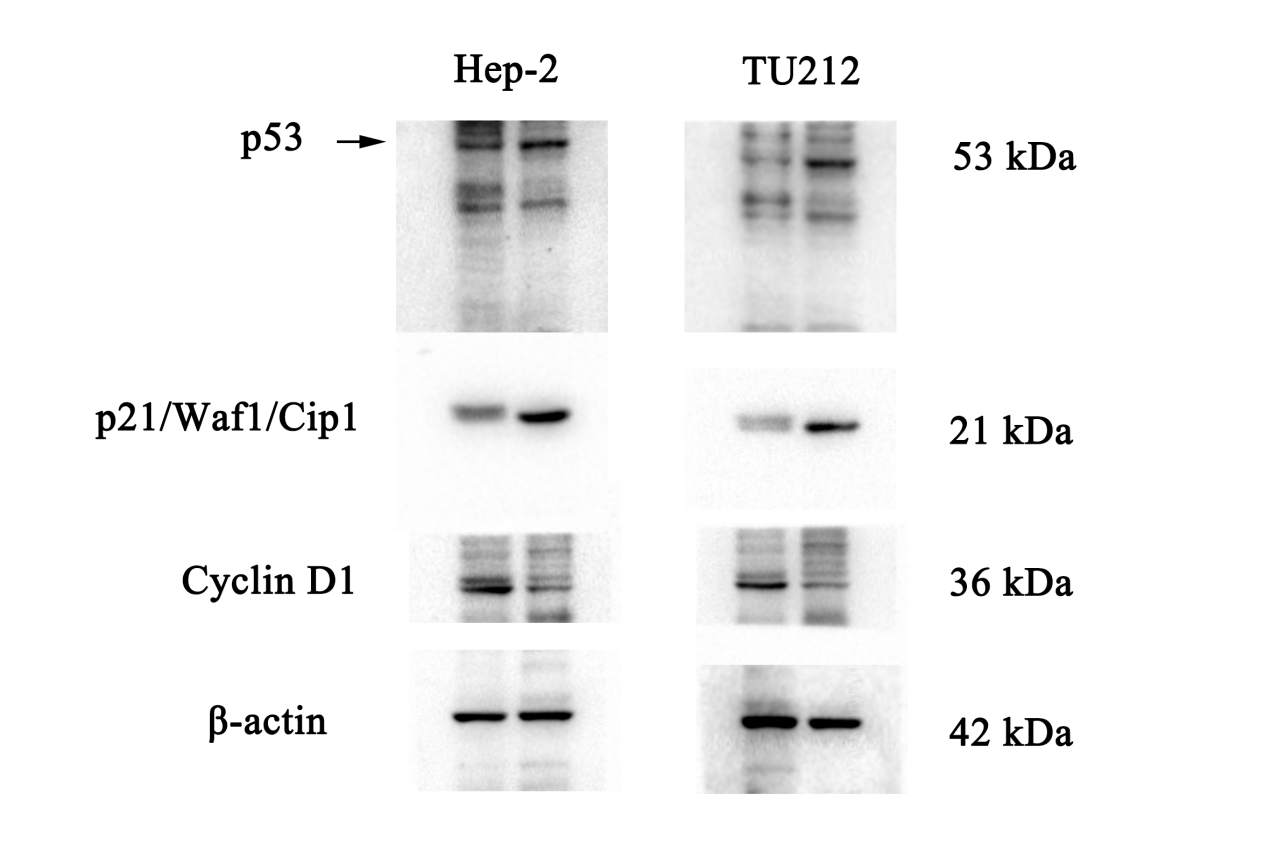


**S6. Original images of Western blotting (Figure 1)**


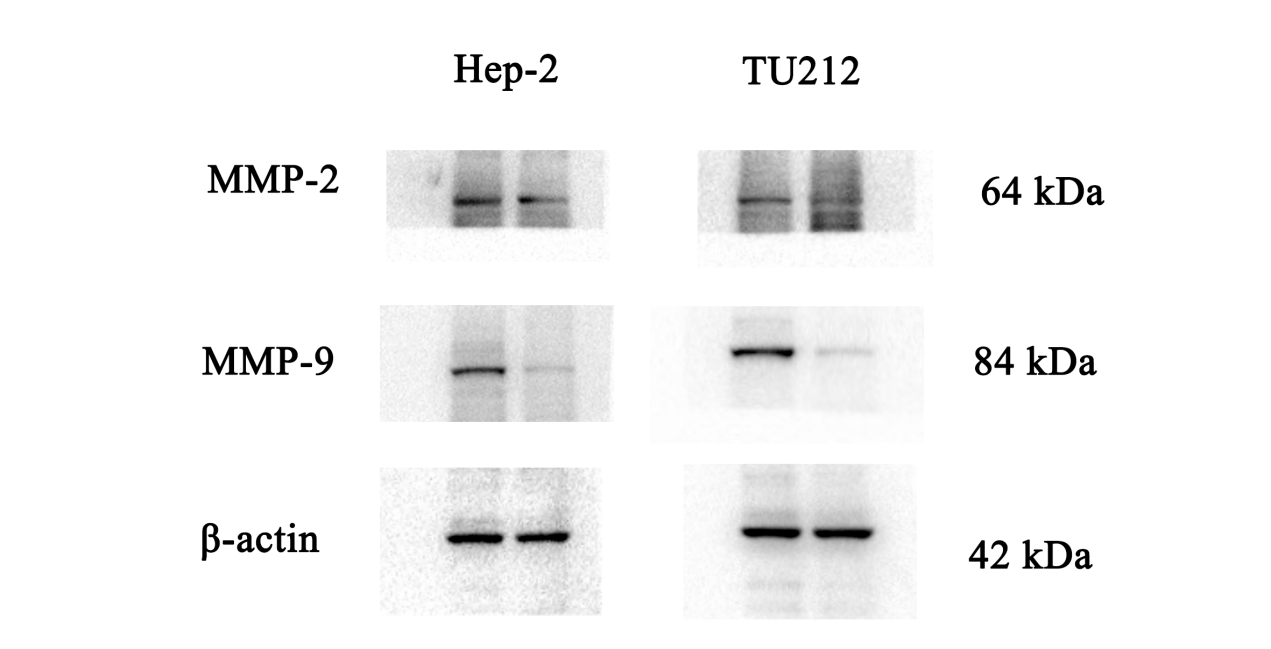


**S7. Original images of Western blotting (Figure 2)**


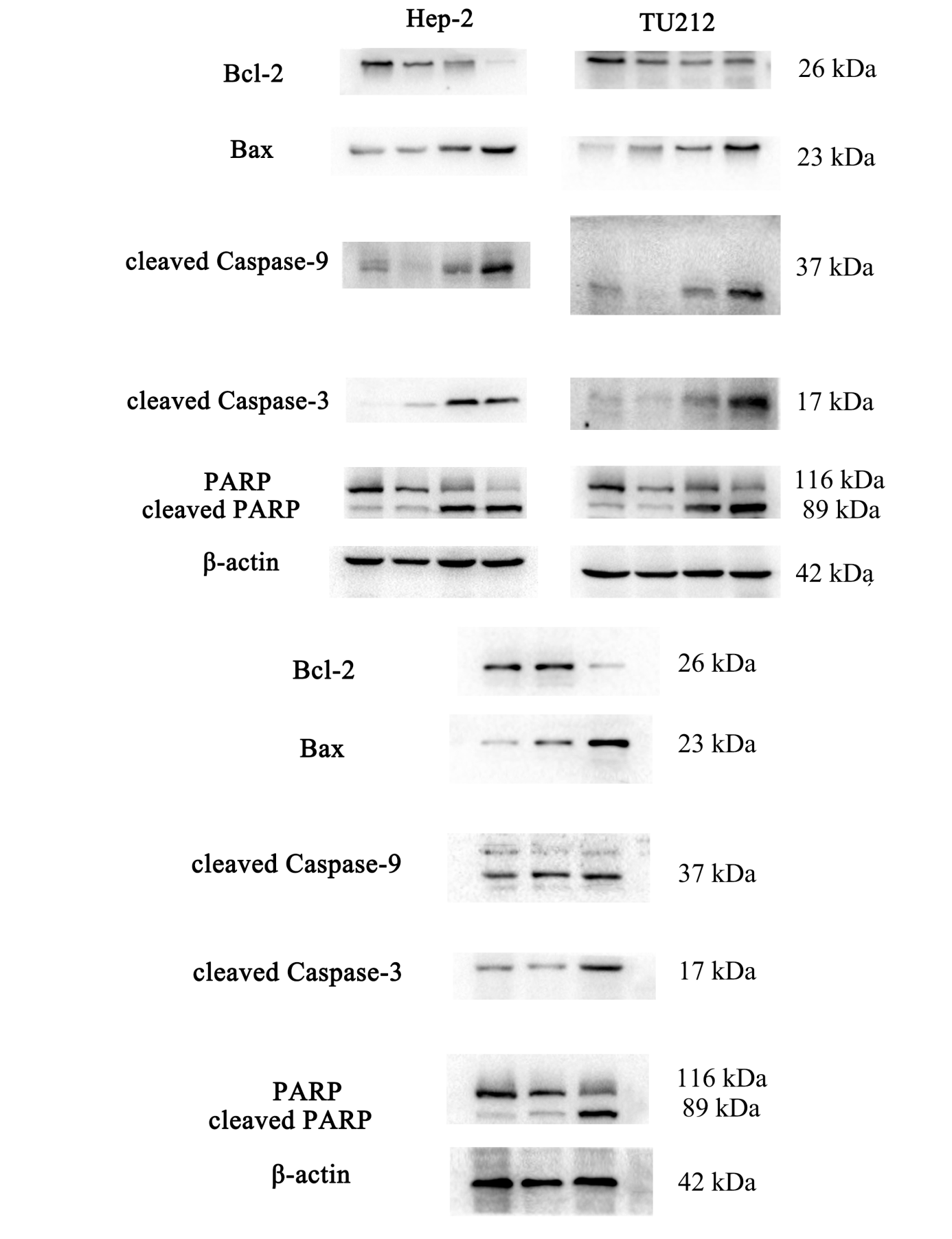


**S8. Original images of Western blotting (Figure 4)**


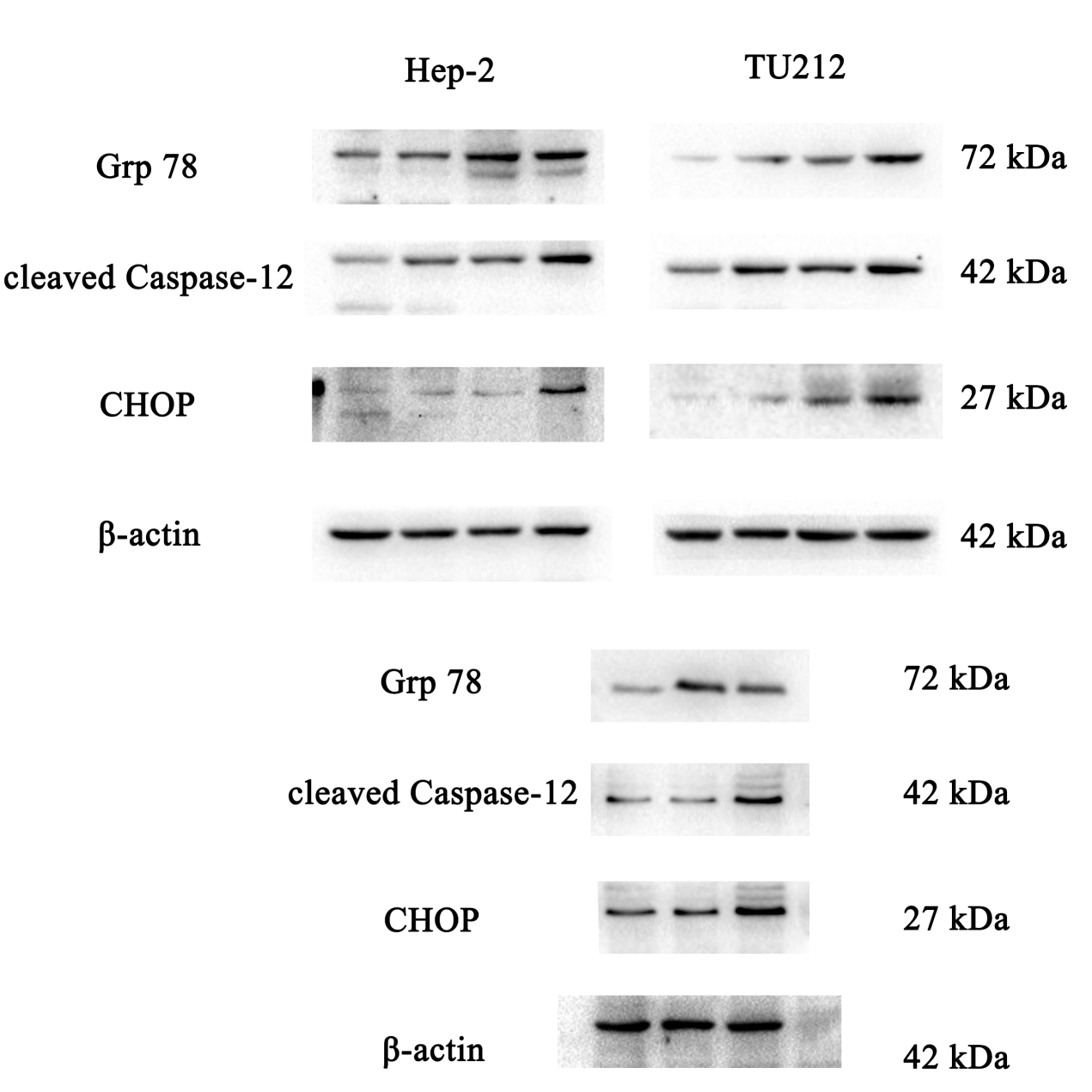


**S9. Original images of Western blotting (Figure 5)**


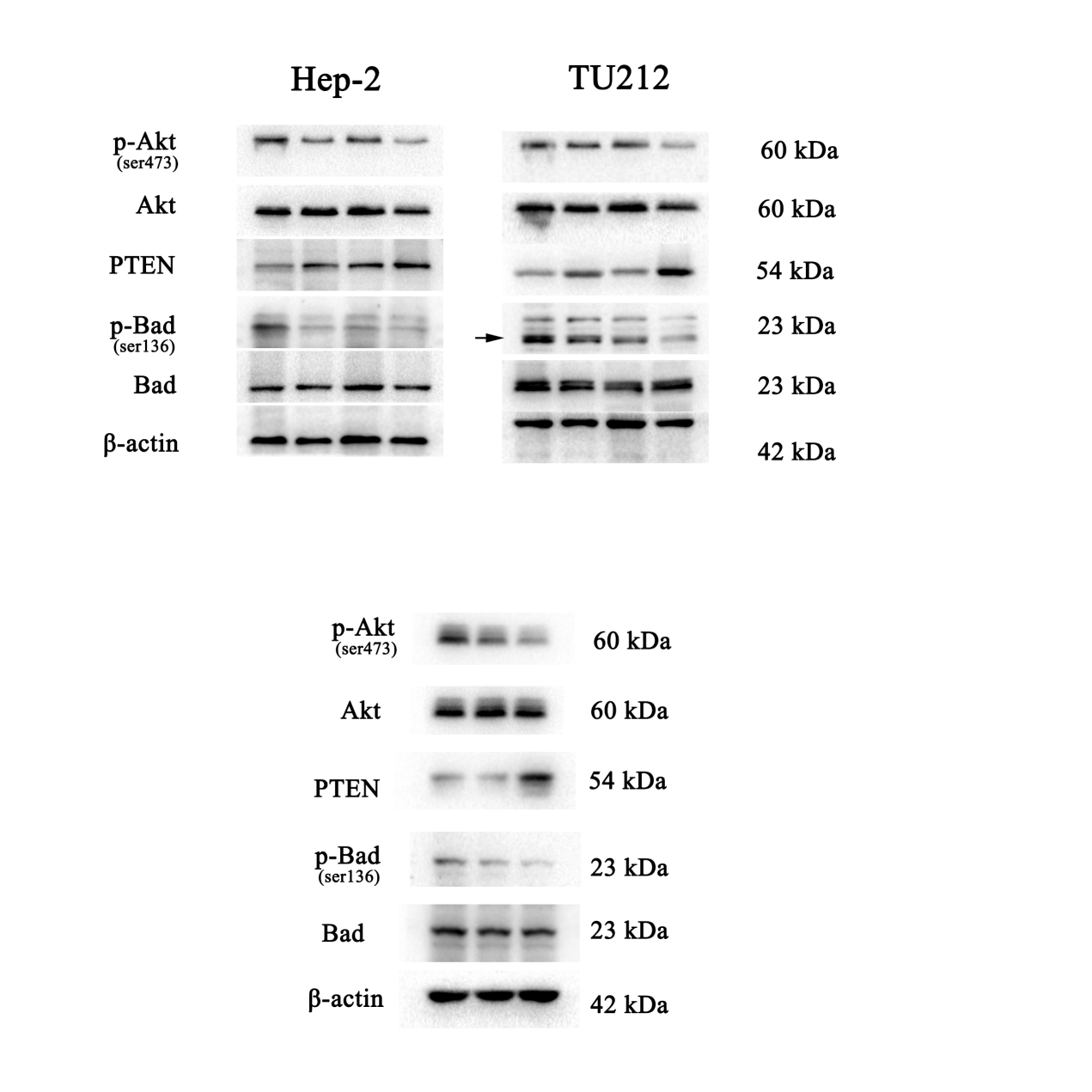


**S10. Original images of Western blotting (Figure 6)**
